# Nursing strategies for the post‐COVID‐19 era

**DOI:** 10.1111/inr.12653

**Published:** 2021-01-06

**Authors:** Jia Lee, Hwa Sook Cho, Sung Rae Shin

**Affiliations:** ^1^ College of Nursing Science Kyung Hee University Seoul Korea; ^2^ Keimyung University Dongsan Medical Center Daegu Korea; ^3^ International Council of Nurses Geneva Switzerland; ^4^ College of Nursing Sahmyook University Seoul Korea

**Keywords:** nursing, nursing strategy, post‐COVID, response

## Abstract

In this indefinite pandemic situation, roles of nurses have been most illuminated by the public, but the nurses may soon start experiencing burnout. This paper highlights roles of nurses in COVID care in Korea and nursing strategies for the post‐COVID era. As we are the frontline advocators of the public, when we develop strategies for their health and quality of life, we receive public support that can help resolve our issues. We will improve our poor working environments through mutual cooperation, as we are one.

**Implications for Nursing:**

Nurses need to possess a variety of abilities, such as the digital literacy required by the non‐contact era after COVID‐19, and to expand the boundaries of the nursing profession in convergence of health services with technology.

## Introduction

Among the currently known pandemics, such as severe acute respiratory syndrome (SARS) from 2002 to 2004 and Middle East respiratory syndrome (MERS) from 2012 to 2013, coronavirus disease 2019 (COVID‐19) has had the biggest impact on the world population. Since the first confirmed case in Korea was reported as a foreign visitor on January 20th, 2020, the number of confirmed cases has increased dramatically, once ranking second worldwide and then declining (The Government of the Republic of Korea 2020), but it is still an uncertain situation that all citizens should be careful of.

### Response strategies in Korea

Response strategies in Korea highlight four characteristics. First, trace, test, and quarantine were the top priorities in infectious disease control. Contact tracing and testing were the most important goals because there have been around 30.8% to 51.7% of asymptomatic cases (Gao et al. 2020). Hence, thermal scanners were deployed to monitor passengers in each building in airports. Self‐quarantine safety applications were promptly developed to manage quarantined cases, including self‐quarantined subject versions as well as assigned case officer versions.

Second, efficient coordination of infrastructures is critical in a pandemic situation, such as medical suppliers, treatment spaces, and the workforce. An infectious disease medical system was proposed for prompt hierarchical response. Central infectious disease hospitals operate the infectious disease control system while regional hub infectious disease hospitals offer treatments in regional areas along with emergency infectious disease centers. Community infectious disease centers are located in public or private hospitals to support vulnerable areas. Infectious disease clinics provide primary care for patients with suspected respiratory infections (Kim 2020).

Third, rapid and safe diagnoses were developed for massive tests. The Korea Center for Disease Control developed test kits with diagnostic manufacturers based on the World Health Organization (WHO) guidelines (The Government of the Republic of Korea 2020). Laboratory tests were conducted at national central laboratories as well as non‐governmental clinical laboratories to cover massive tests. Drive‐through and walk‐through tests were created for safe and efficient screening. The walk‐through test, which is relatively inexpensive, includes a negative pressure booth and a positive pressure booth.

Fourth, transparent communications were imperative for the public to maintain their safety. The Korean government released the number and visiting flow of confirmed cases to the public. The information disclosed includes case number, date, administrative living district, travel history, contact history, and allocated hospital or facility. The government also released concise and easy social distancing guidelines for the public and continued communicating with the public while listening to their opinions. This openness has created trust in the public and makes it possible to identify the areas in which they can contribute and participate. A group of university students developed the Corona Map voluntarily, which was a free smartphone application that offered real‐time information on the visiting flow of confirmed cases (Lee 2020).

## Roles of Nurses

During March 2020, when the number of confirmed cases increased rapidly and more than 90% of them were concentrated in a city located in the southeast of Korea, 3,874 nurses were enlisted as volunteers to move to the city at any time for the COVID care (Korean Nurses Association 2020; Maresca 2020). Roles of nurses in COVID‐19 were positively illuminated from the public. There were five key roles. First, a prompt reorganization of the nursing system was performed to care for COVID patients efficiently. This is because nurses are responsible for managing human and physical infrastructure. The nursing associate director general undertook the general management of nursing while nursing directors managed the nursing staff. Nursing staff were classified as control center nurses, infection control nurses, and ward nurses; their job descriptions were clearly stated (Cho 2020). Through the system, nurses could adjust healthcare spaces to safely provide care by separating clean and infected zones promptly. Additionally, spaces were created between dressing rooms and undressing rooms for personal protective equipment (PPE) and powered air purifying respirators (PAPR).

Second, to improve team communication, nurses directed the accurate documentation and sharing of patients’ conditions among medical staff. Nurses improved communication speed between ward nurses and control center nurses using nursing messengers from electronic medical records (EMRs).

Third, coordinating materials played an important role in emergency situations and continuous care. This is because nurses are closest to the patients and know best what the patients need. Nurses organized, listed, and coded the commonly used intensive care unit (ICU) treatment material sets for easy management.

Fourth, nurses worked to continuously improve the efficiency of healthcare performance as frontline caregivers. In the rapid response team, as nurses monitored patients for 24 h, we were able to detect severe cases early and provide early intervention. Nurses also upgraded electronic medical record system (EMRS), including auto‐documenting medication and vital signs, to save documentation time.

The fifth and last, important role of nurses was caring for other nurses. During this indefinite pandemic situation, nurses may soon start experiencing burnout. It was necessary to monitor nurses and listen to their opinions. A hospital survey of nurses caring for patients with COVID‐19 was conducted from April 24 to May 7, 2020 (Choi 2020). Of the 960 nurses who participated, 38.8% reported working more than two hours with PPE, and most nurses preferred a break time of more than one hour between working with PPE. More than half of the nurses reported presenteeism. Nevertheless, most nurses cited professionalism, followed by partnership, wage or reward, and family as reasons to continue working. Another survey of unfair treatment among 2,489 nurses was conducted from April 27 to May 4, 2020 by the Korean Nurses Association (Shin 2020). About 72.8% of those who participated experienced unfair treatment such as forced shift change, forced individual time off, forced change of work units, and unpaid leave. Based on the data, improvements for a safe working environment and appropriate benefits are important for nurses caring for patients with COVID‐19; however, legal action is sometimes necessary for violating the Labor Standards Act.

## What is Next in Nursing?

**Figure 1:**
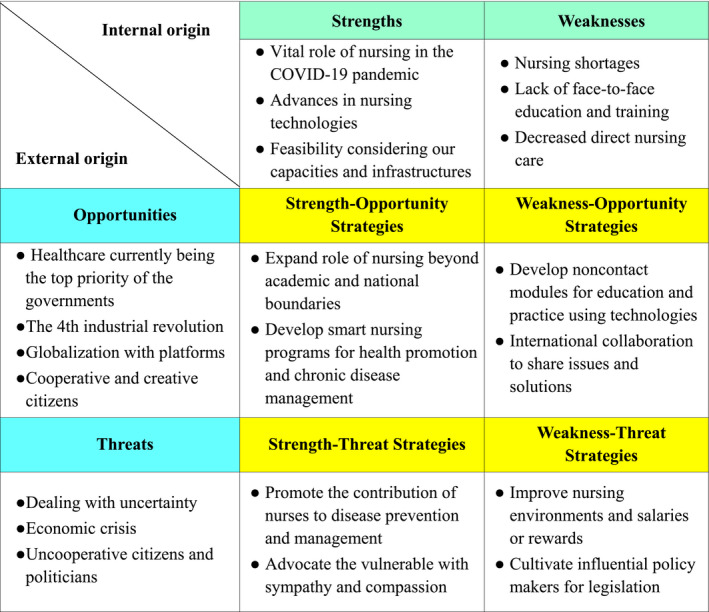
SWOT analysis of nursing for the post‐COVID era.

Figure 1 describes the strength, weakness, opportunity, and threat (SWOT) analysis for nursing in the post‐COVID environment based on a literature review of 46 articles, 102 press news articles, and 33 forums or webinars worldwide. The search strategy included keywords of “coronavirus” or “COVID”, and “care” or “nursing” in PubMed, Google, YouTube, Naver, Daum, and RISS as domestic and international search engines. The authors extracted all themes from the literature published from November 2019 to August 2020 without quality evaluation due to the urgent and rapidly evolving nature of COVID‐19. We assigned these themes to either strengths, weaknesses, opportunities, or threats on the SWOT matrix and then planned each strategies. The strength of nursing includes its vital role in the COVID‐19 pandemic, advances in nursing technologies, and its feasibility considering our capacities and infrastructures; the weaknesses include nursing shortages, lack of face‐to‐face education and training, and decreased direct nursing care.

Opportunities from external sources include health care currently being the top priority of the government, the 4th industrial revolution, and globalization with platforms, while threats from these sources include dealing with uncertainty, economic crisis, and uncooperative citizens and politicians.

### Implications for nursing

The four strategies, in brief, are as follows. First, preparation for the non‐contact era is necessary. As we may need to live with COVID‐19 or other potential infectious diseases until vaccinations are completed, nurses need to possess digital literacy. Information technology (IT), Internet of things (IoT), artificial intelligence (AI), and digital twins will soon be actively used in the nursing field. Smart education and training will be continued and upgraded. We need to create non‐contact care services for health promotion, chronic disease management, and infection control. Nurses should lead and operate new types of healthcare services. During the digital and non‐contact era, ethics and empathy may be negatively affected; as such, each human being should be respected. The treatment of people is more important than making money.

Second, nurses must strive for nursing empowerment considering not only fellow nurses but also the public. For this reason, evidence‐based reports are needed to improve work environments and provide adequate nursing salaries because these are related to the quality of care provided to the public. For nurse leadership, we need to cultivate influential policymakers for legislation to improve the level of public health. We need to advocate for the vulnerable, who are prone to more suffering in such crisis, which is an aspect difficult to overcome. As public relations are important, we also need to continuously promote the contribution of nurses to the prevention and management of diseases as well as in organizing nursing and other personnel using domestic and international media.

Third, we need to expand the boundaries of the nursing profession. In remote health care, nurses ask patients detailed questions about their conditions via video calls through smart phones. The convergence of health services with technology assists nursing staff and increases the accuracy of assessment and monitoring. Nurses can become creators of smart nursing programs using tools such as virtual simulation education programs, AI clinical decision‐making programs, and smartphone apps for health promotion and disease management.

Fourth, global nursing cooperation is the most important in the post‐COVID era. Communities of practice for COVID care will be helpful to overcome future health crises, so the application of lessons learned from these pandemic responses will be very important. Frequent international collaboration to share issues and solutions through platforms is viable. Therefore, the regional and global nursing cooperation team for COVID‐19 can develop a global nursing protocol and an adequate nursing staffing standard for COVID‐19. This cooperative endeavor will be used to support vulnerable countries.

In conclusion, as we are the frontline advocators of the public, when we develop strategies for their health and quality of life, we receive public support that can help resolve our issues. We will improve our work environments through mutual cooperation, as we are one.

## Author contributions

Study design: JL, SS

Data collection: JL, HC

Data analysis: JL, SS, HC

Study supervision: JL

Manuscript writing: JL, SS, HC

Critical revisions for important intellectual contents: JL, SS.
